# ﻿Taxonomic revision of *Camellia* (Theaceae) in Thailand

**DOI:** 10.3897/phytokeys.239.113878

**Published:** 2024-03-05

**Authors:** Dongwei Zhao

**Affiliations:** 1 Department of Forestry, College of Forestry, Central South University of Forestry and Technology, Changsha, Hunan 410004, China Central South University of Forestry and Technology Changsha China

**Keywords:** Assam tea, Indochina, new records, oil camellias

## Abstract

Natural plants of *Camellia* (Theaceae) in Thailand are taxonomically revised with a key, a distribution map, descriptions, specimens examined, and figures of living plants and/or dry specimens. Nine taxa comprising seven species and two varieties are recognized, including *C.caudata*, *C.connata*, *C.furfuracea*, *C.kissi*, C.kissivar.confusa, *C.laotica*, C.sinensisvar.assamica, *C.suddeeana*, and *C.taliensis*. *Camelliacaudata* and *C.laotica* are new records to Thailand, and *C.connata* and *C.suddeeana* are endemic to the country. Formerly recorded *C.pleurocarpa* and *C.tenii* are excluded from Thai flora because of misidentification, and *C.oleifera* and *C.sinensis* are merely cultivated in the country. Morphological descriptions of *C.connata* and *C.laotica* are improved based on additional collections examined.

## ﻿Introduction

*Camellia* L. (Theaceae) contains economically important plants, including tea, oil camellia and camellias. They are evergreen shrubs or trees distributed in East, South and Southeast Asia (Zhao et al. 2023). Plants of *Camellia* generally bear a smooth bark, simple and alternate leaves without stipules, coriaceous leaf blades, axillary and bisexual flowers, dorsifixed anthers, a superior ovary, a loculicidal capsule with a columella and wingless seeds. Sealy (1958) recorded 82 species and 24 doubtful names in his monograph of *Camellia*. Chang (1998) counted about 280 species in the genus but Ming (2000) revised the number of species into 119. Since 2000, more than 100 species have been described in *Camellia* (e.g., Orel and Curry 2015, 2019; Liu et al. 2020; Ye et al. 2022; Zhao 2023). However, previous monographers of *Camellia*, such as Sealy (1958), Chang (1981, 1998) and Ming (2000), generally focused on the species distributed in China but hardly examined the collections at local herbaria in Indochina (Zhao 2022a). For example, the specimens of *Camellia* at Thai herbaria have not been thoroughly inspected since Keng’s (1972) work.

Craib (1925) recognized three species of *Camellia* for Thailand, including *C.confusa* (Craib) Cohen-Stuart, *C.connata* (Craib) Craib and *C.theifera* Griff., in his “Florae Siamensis Enumeratio”. Keng (1972) subsequently recorded six taxa of the genus, viz. *C.connata*, C.oleiferaC. Abelvar.confusa (Craib) Sealy, *C.pleurocarpa* (Gagnep.) Sealy, C.sinensis(L.)Kuntzevar.assamica (Royle ex Hook.) Steenis (Assam tea), *C.taliensis* (W.W. Sm.) Melch. and *C.tenii* Sealy, in “Flora of Thailand”. Later, Ming (2000) and Zhao (2021) added *C.kissi* Wall. and *C.furfuracea* (Merr.) Cohen-Stuart to the Thai flora, respectively. Zhao (2023) described a new species, *C.suddeeana* D.Wei Zhao from Thailand. However, Thailand is a botanically under-collected country (Parnell et al. 2003). Here I present a taxonomic revision for *Camellia* in Thailand based on taxonomic literature (Craib 1925; Sealy 1958; Keng 1972; Smitinand 1975; Chang 1981; Gardner et al. 2000; Ming 2000; Maxwell and Elliott 2001; Zhao et al. 2023), herbarium specimens, and fieldwork performed by myself. Two new records are reported along with an up-to-date taxonomic revision of *Camellia* for the country.

## ﻿Material and methods

Morphological characters were described based on living plants, herbarium specimens or their images from BK, BKF, BM, C, CMUB, E, GXMI, HITBC, IBK, IBSC, K, KKU, KUN, L, MO, NSW, P, PE, PNH, QBG, SING, SYS, and TCD (acronyms following Thiers 2024, continuously updated). Geographic distribution data were retrieved from the specimen records and mapped using ArcMap 10.7 (ArcGIS, ESRI) and optimized in Adobe Illustrator CS3. All photos of dry specimens and living plants in the figures were taken and adapted by the author.

## ﻿Results

A key to all nine taxa of *Camellia* occurring in Thailand is present below. Taxa in Thailand are subsequently listed alphabetically.

### ﻿Key to taxa of *Camellia* in Thailand

**Table:** 

1	New branchlet and petiole glabrous	**2**
–	New branchlet and petiole hairy	**3**
2	Pedicel 1–3 mm long, sepals abaxially pubescent	***C.furfuracea***
–	Pedicel 4–8 mm long, sepals abaxially glabrous	***C.taliensis***
3	Sepals caducous after flowering	**4**
–	Sepals persistent after flowering	**5**
4	Flowers 1–4.5 cm in diam., styles 1.5–7 mm long, pericarp 0.5–1.5 mm thick	***C.kissi***
–	Flowers 4.5–10 cm in diam., styles 8–12 mm long, pericarp 2–8 mm thick	**C.kissivar.confusa**
5	Style 1, apically (2–)3–5 lobed	**6**
–	Styles 3, distinct	**8**
6	Sepals 3–4, ovary glabrous	***C.laotica***
–	Sepals 5, ovary hairy	**7**
7	Bracteoles caducous, sepals abaxially glabrous	**C.sinensisvar.assamica**
–	Bracteoles persistent, sepals abaxially pubescent	***C.caudata***
8	Filaments yellowish orange, completely united into a tube, ovary pubescent	***C.connata***
–	Filaments white, basally connate ca. 1/3, ovary glabrous	***C.suddeeana***

#### ﻿1. Camellia caudata

Taxon classificationPlantaeEricalesTheaceae

Wall., Pl. Asiat. Rar. 3: 36. 1832.

CF78C450-FB9A-5EEB-BD57-D22857A40C03

= Camelliaassimilis Champ. ex Benth., Hooker’s J. Bot. Kew Gard. Misc. 3: 309. 1851. Lectotype (designated by Chang & Bartholomew [1984: 205]): China. Hong Kong, *J.G. Champion 65* (K 000380537!).
= Camelliagracilis Hemsl., Ann. Bot. (Oxford) 9(33): 146. 1895. Holotype: China. Taiwan: Bankinsing mountains, *A. Henry 1612* (K 000380535!).
= Camelliatriantha Hung T. Chang, Taxon. Gen. Camellia 144. 1981. Holotype: China. Guangxi: Yongning, 20 October 1963, *F.S. Huang 17625* (SYS 00094835!).
= Camelliatubiformis Hung T. Chang & S.X. Ren, Acta Sci. Nat. Univ. Sunyatseni 31(1): 75. 1992. Holotype: China. Guangdong: Fengkai, Heishiding, 580 m, 1 January 1991, *R.X. Jiang s.n.* (SYS 00094839!).

##### Type material.

***Lectotype*** (designated by Zhao et al. [2017a: 172]): India. [Meghalaya: Khasia Hills], the district of Sylhet, November 1827, *H. Bruce s.n.* in *N. Wallich 978* (right-hand specimen of K 001110475!).

##### Description.

**Figure 1.**
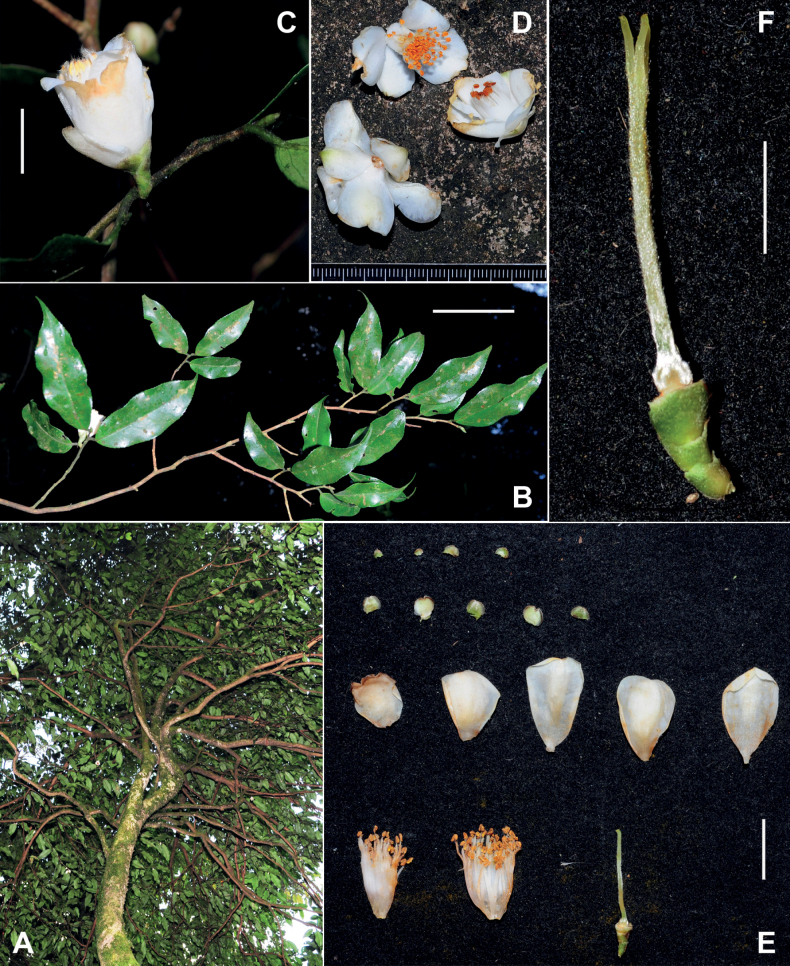
*Camelliacaudata***A** canopy of a tree **B** branch **C** flower **D** petals and androecia **E** a dissected flower **F** gynoecium and pedicel. Scale bars: 5 cm (**B**); 1 cm (**C, E**); 5 mm (**F**). The minimum graduation of the ruler in **D** indicates 1 mm.

Trees or shrubs up to 15 m tall. ***New branchlets*** and ***terminal buds*** pubescent. ***Petioles*** 1–5 mm long, pubescent; ***leaf blades*** elliptic, oblong or lanceolate, 3.5–11.5 × 1.5–3 cm, thinly coriaceous or papery, abaxially pale green, sparsely appressed pubescent or villous, adaxially yellowish or dark green, shiny, hirsute along midrib, midrib abaxially elevated and adaxially slightly impressed, secondary veins 8–12 on each side of midrib, abaxially slightly elevated and adaxially obscure, base attenuate to obtuse, margin serrulate, apex caudate. ***Flowers*** solitary or up to 3 in a cluster, 2–4 cm in diam. ***Pedicel*** 2.5–7 mm long. ***Bracteoles*** 3–6, alternate, persistent, deltate to semi-orbicular, 1–2.5 × 1–3 mm, abaxially sparsely pubescent to pubescent, adaxially glabrous, margin ciliolate. ***Sepals*** 5, persistent, suborbicular, 2–3 × 2–4 mm, abaxially pubescent, adaxially glabrous. ***Petals*** 5–7 in 1–2 whorls, white, elliptic to obovate, 11–20 × 8–14 mm, abaxially pubescent, adaxially glabrous, apex rounded to emarginate, inner 4–5 petals basally adnate to filament whorl for 2–4 mm. ***Stamens*** numerous, 10–15 mm long; filaments white, outer filaments basally connate for 6–10 mm, distinct part villous. ***Ovary*** globose to ovoid, densely pubescent. ***Style*** 1, 8–18 mm long, basally densely pubescent and gradually becoming sparsely pubescent apically, apically 3-lobed for 1–3 mm. ***Capsule*** globose to ovoid, 11–15 mm in diam., 1-loculed with 1 seed; pericarp 0.4–1 mm thick. ***Seeds*** fuscous, globose, 1–1.5 cm in diam., glabrous Fig. 1.

##### Phenology.

Flowering October–December, fruiting March–December.

##### Distribution and habitat.

**Figure 2.**
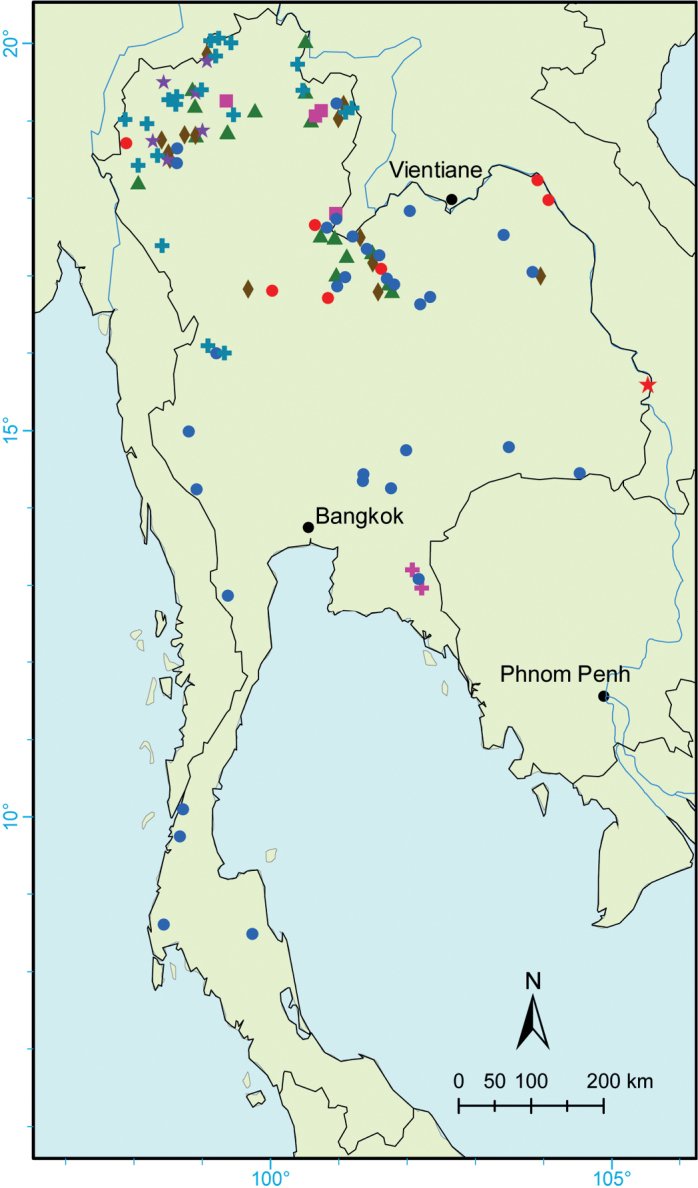
Distribution of *Camellia* taxa in Thailand: *C.caudata* (Pink square), *C.connata* (Purple star), *C.furfuracea* (Pink plus), *C.kissi* (Blue dot), C.kissivar.confusa (Brown diamond), *C.laotica* (Red star), C.sinensisvar.assamica (Green triangle), *C.suddeeana* (Red dot) and *C.taliensis* (Blue plus).

*Camelliacaudata* occurs in semi-evergreen, evergreen and deciduous montane forests at the elevations of 500–2000 m in China, India, Laos, Myanmar, Nepal, Thailand (Fig. 2), and Vietnam.

##### Additional specimens examined.

**Chiang Rai**: Wiang Pa Pao, Doi Luang, Doi Mok Mountain, close to source of Man Mae Nga Stream, 19°15'36"N, 99°20'24"E, 950 m, 23 July 1998, *Morci 1365.0* (CMUB).

**Nan**: Doi Tiu, 1100 m, 8 March 1921, *Kerr 5041* (BK 203924, BM, K, L.2399739); Tha Wang Pha, Pa Kha, Doi Wao, 1400–1700 m, 10 September 1995, *Larsen et al. 46319* (BKF SN147973) & *46292* (IBSC 0256482).

**Uttaradit**: Phu Soi Dao, 1531 m, 17 November 2009, *Norsaengsri & Intamusik 6104* (QBG 42621), 1570 m, 18 November 2009, *Norsaengsri & Intamusik 6202* (QBG 42737).

##### Notes.

Wallich (1829) provided a *nomen nudum* of *C.caudata*, the name was subsequently validated in Wallich (1832). In the protologue of *C.triantha*, Chang (1981: 146) stated that the filaments, styles and ovaries of the species were glabrous and indicated the holotype at SYS. The holotype consists of a single leaf and two dissected flower buds upon which Chang’s (1981: 146, 1998: 159) descriptions were generally based. However, two isotypes of *C.triantha* (GXMI 050183, GXMI 050184) with mature flowers clearly show that the filaments, styles and ovaries of the species are not glabrous but densely pubescent. Therefore, I agree with Ming’s (2000: 201) view and treat *C.triantha* as a heterotypic synonym of *C.caudata*.

*Camelliacaudata* is a new record to Thailand. It generally occurs in the montane forests at elevations of 900–1700 m of Northern Thailand. The species is one of several camellias that are widely distributed in Indochina, along with Assam tea, *C.furfuracea* and *C.kissi*. Kerr A.F.G. collected specimens of *C.caudata* in 1921 (*Kerr 5041* at BK, BM, K and L). The specimens were, however, misidentified as *C.connata* by Keng H. in 1970 as shown on the sheet conserved at BM. *Camelliacaudata* can be distinguished from *C.connata* by its basally connate styles whereas the latter bears distinct styles.

#### ﻿2. Camellia connata

Taxon classificationPlantaeEricalesTheaceae

(Craib) Craib, Fl. Siam. 1(1): 131. 1925.

6A7BD0A4-D020-5832-A268-8C6C2DCABA66

≡ Theaconnata Craib, Bull. Misc. Inform. Kew (1): 6. 1914. Lectotype (first-step designated by Sealy [1958: 146]; second-step designated by Zhao et al. [2017a: 174]): Thailand. Chiang Mai: Doi Suthep, 1520 m, 25 June 1911, *A.F.G. Kerr 1878* (K 000704325!).

##### Description.

**Figure 3.**
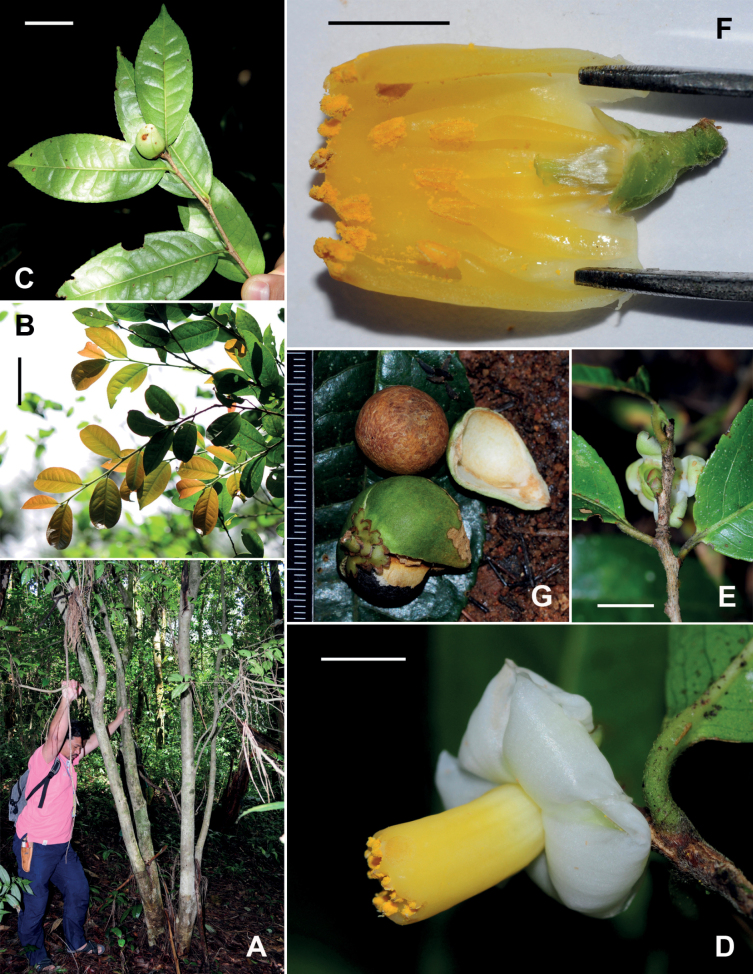
*Camelliaconnata***A** habit **B** branchlets **C** a branch with an immature fruit **D, E** flowers **F** a dissected flower without corolla **G** a fruit and seeds. Scale bars: 5 cm (**B**); 2 cm (**C**); 5 mm (**D, F**); 1 cm (**E**). The minimum graduation of the ruler in **G** indicates 1 mm.

Shrubs or trees up to 8 m tall. ***New branchlets*** puberulous; ***terminal buds*** pubescent. ***Petioles*** 2.5–7 mm long, sparsely puberulous; ***leaf blades*** elliptic to oblong, 5.5–12 × 3–5 cm, thinly coriaceous, abaxially yellowish green and brown punctate, glabrous or sparsely puberulous along midrib, adaxially yellowish or dark green, shiny, hirsute along midrib, midrib abaxially elevated and adaxially slightly raised, secondary veins 9–12 on each side of midrib, slightly raised on both surfaces, base attenuate to rounded, margin serrulate, apex acute to attenuate. ***Flowers*** solitary or paired, ca. 2 cm in diam., subsessile. ***Bracteoles*** 3–6, alternate, persistent, gradually transitioning to sepals, deltate to semi-orbicular, 1.5–3 × 1–2.5 mm, abaxially glabrous or sparsely puberulous, adaxially glabrous. ***Sepals*** 4–5, persistent, sub-orbicular or ovate, 3.5–5 × 4–6 mm, abaxially glabrous or sparsely puberulous, adaxially glabrous, margin ciliolate and usually lacerate. ***Petals*** 6–7 in 2 whorls, elliptic or ovate, glabrous, outer 2 petals 5–7 × 7–9 mm, greenish white, inner 4–5 petals basally adnate to filament whorl for ca. 3 mm. ***Stamens*** 14–20; filaments yellowish orange, glabrous, outer 9–14 filaments completely united into a 7–12 mm long tube, ca. 5 mm in diam., apex irregularly dentate, each tooth bearing an anther, remaining filaments 6–9 mm long, borne on the inside of filament tube. ***Ovary*** globose to ovoid, pubescent. ***Styles*** 3, distinct, ca. 1.5 mm long, glabrous or sparsely puberulous at base. ***Capsule*** ovoid or globose, 10–25 mm in diam., 1–3-loculed with 1–3 seeds; pericarp 0.5–1 mm thick. ***Seeds*** black to brown, globose or hemispherical, 1–1.5 cm in diam., glabrous Fig. 3.

##### Phenology.

Flowering April–August, fruiting July–November.

##### Distribution and habitat.

*Camelliaconnata* is endemic to northern Thailand (Fig. 2), in the evergreen, mixed evergreen and deciduous forests at the elevations of 550–2000 m.

##### Additional specimens examined.

**Chiang Mai**: 610 m, 16 July 1923, *Winit 1134* (BKF SN060818, K); Chiang Dao, Doi Chiang Dao, east side of Sop Huay Pah Dahng-Huay Nah Lao Station, 575 m, 18 August 1995, *Bella & Nanny 5* (CMUB 06549); Chom Thong, Doi Inthanon, Mae Uam Station, ca. 1700 m, 2 April 2008, *Watthana 2735* (QBG 37391); Fang, Doi Ang Khang, ca. 1500 m, 14 July 1922, *Kerr 6294* (BK 203711, BM, K), 1400–1800 m, 8 June 1976, *Charoensorn s.n.* (BK 203714); Mae Chaem, ca. 800 m, 22 October 1922, *Kerr 6428* (BK 203710, K), Doi Inthanon, between 34 km and 35 km, on the road to Mae Aum Watershed Management Station, 1590–1640 m, 12 November 2015, *Zhao et al. 84* (BKF, TCD), *85–88* (TCD), *89* (BKF, TCD), *90* (BKF, KUN, TCD), *91* (KUN, TCD), *92* (BKF, KUN, TCD), *93 (1), (2)* (TCD) & *94* (BKF, KUN, TCD); Mueang Chiang Mai, Doi Suthep-Pui, 18°49'42"N, 98°53'26"E, 1636 m, 13 November 2015, *Zhao et al. 97 (1) & (2)* (TCD). **Mae Hong Son**: Pai, Doi Chang, 2000 m, 31 May 1977, *Santisuk 1131* (BKF SN178498).

##### Notes.

Cohen-Stuart (1916, 1919) established Camelliasect.Calpandria (Blume) Cohen-Stuart based on *C.lanceolata* (Blume) Seem. and thought that *T.connata* might be a heterotypic synonym of *C.lanceolata*. Sealy (1958) suggested that the two species were distinct from each other and placed *C.connata* in sect. Calpandria because both plants bore a filament tube. Subsequent monographers of *Camellia*, including Chang (1981), Chang and Bartholomew (1984) and Ming (2000), agreed with the treatment of Sealy (1958). However, phylogenetic analysis (Zhao et al. 2023) suggested that *C.connata* was nested in the clade of Heterogenea and did not have a close relationship with *C.lanceolata*, which implies that the filament tube is not a synapomorphy in *Camellia*. The descriptions of *C.connata* provided in Sealy (1958) and Ming (2000) were based on several specimens collected by Kerr and Winit about a century ago. A detailed account of *C.connata* is supplied here with recent collections and photos of living plants (Fig. 3).

#### ﻿3. Camellia furfuracea

Taxon classificationPlantaeEricalesTheaceae

(Merr.) Cohen-Stuart, Bull. Jard. Bot. Buitenzorg 1(4): 240. 1919.

FD7A2579-DC18-5BD6-A832-39EF05FBB7D9

≡ Theafurfuracea Merr., Philipp. J. Sci., C 13: 149. 1918. Holotype: China. Guangdong: Huizhou, Boluo, Luofu Mountain, 9–27 August 1917, *E.D. Merrill 10681* (PNH 87432, image!).
= Theabolovenensis Gagnep., Notul. Syst. (Paris) 10: 124. 1942. Lectotype (designated by Ming [2000: 225]): Laos. Champasak: Plateau des Boloven, entre Nong Bok Kao et Phong Tham, 900 m, 6 October 1928, *E. Poilane 15856* (K 000704324!).
= Camelliasuaveolens C.X. Ye, X.J. Wang & X.G. Shi, Acta Sci. Nat. Univ. Sunyatseni 43(3): 129. 2004. Holotype: China. Guangdong: Yingde, cultivated, introduced from Lechang, 27 October 2002, *C.X. Ye 5919* (SYS 00142796!).
= Camelliamaiana Orel, Novon 20(2): 198. 2010. Holotype: Vietnam. Lam Dong: Dalat Plateau, 19 November 2002, *G. Orel et al. 21149* (NSW 901884, image!).
= Camelliacurryana Orel & Luu, Nordic J. Bot. 32(1): 42. 2014. Holotype: Vietnam. Lam Dong: Dalat Plateau, 27 February 2002, *G. Orel et al. 21147* (NSW 901031, image!).
= Camelliaduyana Orel, Curry & Luu, Novon 23(3): 308. 2014. Holotype: Vietnam. Lam Dong: Dalat Plateau, ca. 1400 m, 27 November 2010, *G. Orel & N.V. Duy 0719* (NSW 901883, image!).
= Camelliaalbata Orel & Curry, Pursuit Hidden Camellias Vietnam China 239. 2015. Holotype: Vietnam. Quang Ninh, 2 December 1999, *G. Orel et al. 991202c* (NSW 901898 [sheet 1 of 2, image!] and NSW 849513 [sheet 2 of 2, image!]).
= Camelliareflexa Orel & Curry, Pursuit Hidden Camellias Vietnam China 209. 2015. Holotype: Vietnam. Vinh Phuc: Tam Dao National Park, 2 April 2001, *G. Orel et al. 1240* (NSW 901749, image!).
= Camelliaviscosa Orel & Curry, Pursuit Hidden Camellias Vietnam China 214. 2015. Holotype: Vietnam. Lam Dong: Dalat Plateau, 19 November 2002, *G. Orel et al. 21148G* (NSW 901821, image!).

##### Description.

**Figure 4.**
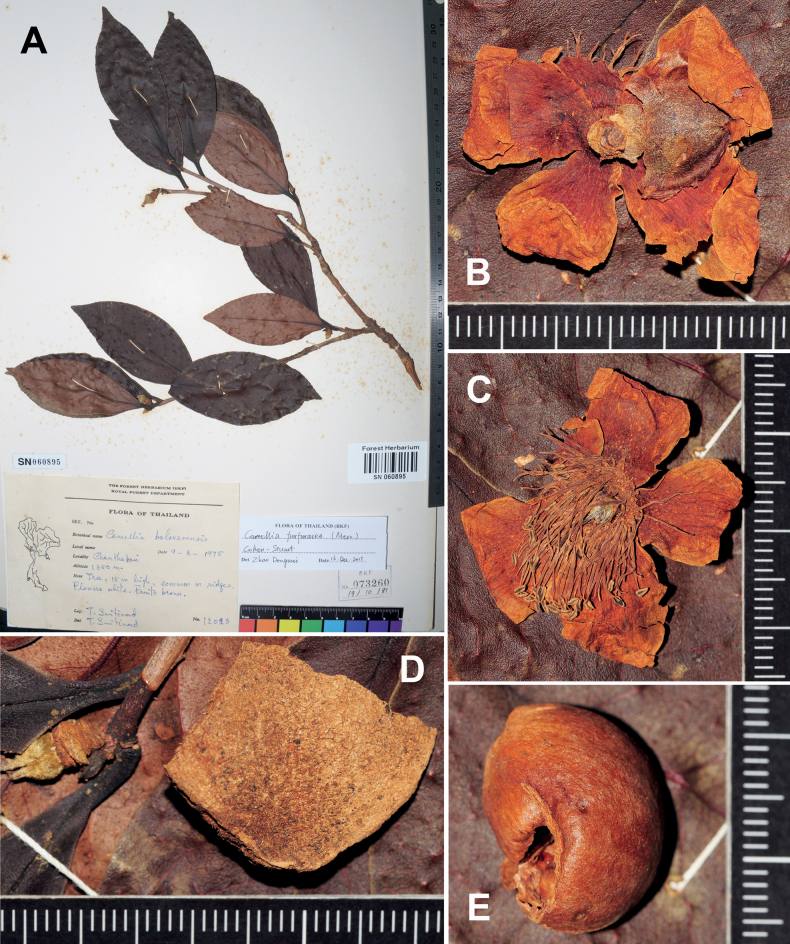
Dry specimens of *Camelliafurfuracea*, *Smitinand 12023* at BKF **A** specimen sheet **B, C** flower fragments **D** a part of pericarp along a branchlet bearing a pedicel and gynoecium remains **E** a broken seed. The minimum graduation of the rulers represents 1 mm.

Shrubs or trees up to 15 m tall. ***New branchlets*** glabrous; ***terminal buds*** glabrous or puberulous. ***Petioles*** 3–12 mm long, glabrous; ***leaf blades*** elliptic or oblong, 7.5–17 × 3–7 cm, coriaceous, abaxially yellowish or pale green, brown punctate or glabrous, adaxially dark green, glabrous and shiny, midrib abaxially elevated and adaxially channelled, secondary veins 7–11 on each side of midrib, abaxially raised and adaxially slightly impressed, base attenuate, cuneate or obtuse, margin sparsely serrulate, apex attenuate, acute or obtuse. ***Flowers*** solitary or paired, 4–6 cm in diam. ***Pedicels*** 1–3 mm long. ***Bracteoles*** and ***sepals*** 6–9, caducous, crescent or ovate, 2–13 × 3.5–20 mm, abaxially puberulous or pubescent, adaxially glabrous, margin membranous. ***Petals*** 6–9 in 1–2 whorls, white, elliptic or obovate, 17–25 × 9–15 mm, abaxially puberulous or glabrous, adaxially glabrous, basally connate for 1–2 mm. ***Stamens*** numerous, 10–15 mm long; filaments glabrous, basally adnate to petals for ca. 2 mm. ***Ovary*** ovoid or globose, pubescent. ***Styles*** 3–4, distinct, 6–10 mm long, pubescent and gradually becoming glabrous apically. ***Capsule*** oblate or globose, 3–5 cm in diam., 2–3 cm in height; usually 3-loculed with 1–3 seed per locule; pericarp 1–10 mm thick, furfuraceous. ***Seeds*** brown or fuscous, hemispherical or polyhedral, 1.5–2 cm in diam., glabrous Fig. 4.

##### Phenology.

Flowering October–February, fruiting December–April.

##### Distribution and habitat.

*Camelliafurfuracea* is distributed in the evergreen forest, on slopes or along streams at elevations of 450–1800 m in China, Laos, Thailand (Fig. 2), and Vietnam.

##### Additional specimens examined.

**Chanthaburi**: Pong Nam Ron, Khao Soi Dao, ca. 1600 m, 23 January 1956, *Smitinand 3242* (BKF SN060898), 1100 m, 5 April 1974, *Smitinand 11994* (BKF SN060894), 1650 m, 9 February 1975, *Smitinand 12023* (BKF SN060895, BKF SN060896, P 04500286; Fig. 4); Soi Dao, Khao Soi Dao, 1300–1400 m, 12 December 1924, *Kerr 9638* (BK 203925, BM, C, K, P 04500307).

##### Notes.

*Camelliafurfuracea* is absent in Keng (1972). A specimen of *C.furfuracea* collected in 1956, *Smitinand 3242* at BKF, was misidentified as C.oleiferavar.confusa by Keng in 1970. Zhao (2021) recognized six heterotypic synonyms of *C.furfuracea* and referenced specimens from Thailand (listed above), which made the species a new record to the country. *Camelliafurfuracea* widely occurs in subtropical and tropical China and Indochina. It can be distinguished from C.kissiWall.var.confusa (Craib) T.L. Ming by its glabrous new branchlets, usually abaxially punctate leaves and apically divided ovary with distinct styles (Fig. 4), whereas the latter bears puberulous new branchlets, abaxially glabrous leaves and basally connate styles.

#### ﻿4. Camellia kissi

Taxon classificationPlantaeEricalesTheaceae

Wall., Asiat. Reschs. 13: 429. 1820.

F5C503E4-CB7F-5E4D-BA59-AEFD3BB55ED4

= Camelliakeina Buch.-Ham. ex D. Don, Prodr. Fl. Nepal. 224. 1825. Holotype: Nepal. Narain hetty, 9 September 1802, *F. Buchanan-Hamilton s.n.* (BM 000521691!).
= Camelliasymplocifolia Griff., Itin. Pl. Khasyah Mts. 40, No. 652. 1848. Lectotype (designated by Zhao [2022b: 16]): India. Khasya Hills, *W. Griffith s.n.* (TCD 0018254!).
= Thea bachmaensis Gagnep., Notul. Syst. (Paris) 10: 124. 1942. Lectotype (first-step designated by Sealy [1958: 199]; second-step designated by Zhao et al. [2017a: 173]): Vietnam. [Thua Thien Hue]: Núi Bach Ma Station d’altitude de Huê, 1400–1500 m, 12 December 1940, *E. Poilane 31118* (P 01903389! Image: https://science.mnhn.fr/institution/mnhn/collection/p/item/p01903389).
= Thea brachystemon Gagnep., Notul. Syst. (Paris) 10: 125. 1942. Lectotype (designated by Zhao et al. [2017a: 173]): Laos. [Attapeu]: entre Nong Met et B. Thuôt, plateau des Boloven, 8 October 1928, *E. Poilane 15908* (P 01903386! Image: https://science.mnhn.fr/institution/mnhn/collection/p/item/p01903386).
= Theopsiseuonymifolia Hu, Acta Phytotax. Sin. 10(2): 140. 1965. Holotype: China. Yunnan: Pu’er, Jingdong, 1050 m, 13 December 1939, *M.G. Li 1506* (KUN 1206027!).
= Camelliathailandica Hung T. Chang & S.X. Ren, Acta Sci. Nat. Univ. Sunyatseni 30(1): 67. 1991. Holotype: Thailand. Khao Yai National Park, Khao Khieo, 14°21'N, 101°22'E, 1200–1300 m, 29 October 1970, *C. Charoenphol et al. 4205* (MO; isotypes BKF!, C!, K!).
= Camellialigustrina Orel, Curry & Luu, Novon 23(3): 310. 2014. Holotype: Vietnam. Lam Dong: Mount Lang Biang, 1850 m, 16 December 2011, *G. Orel & A.S. Curry 0734* (NSW 900397, image!).
= Camelliacuongiana Orel & Curry, Pursuit Hidden Camellias Vietnam China 180. 2015. Holotype: Vietnam. Lam Dong: Bidoup-Nui Ba National Park, 5 November 2012, *G. Orel et al. 0721* (NSW 901040, image!).

##### Type material.

***Holotype***: NEPAL. 1818, *Gardner s.n.* (BM 000948697!).

#### ﻿4a. Camellia kissi var. kissi

Taxon classificationPlantaeEricalesTheaceae

A1336350-5782-523F-9959-CCDD40D2648A

##### Description.

**Figure 5.**
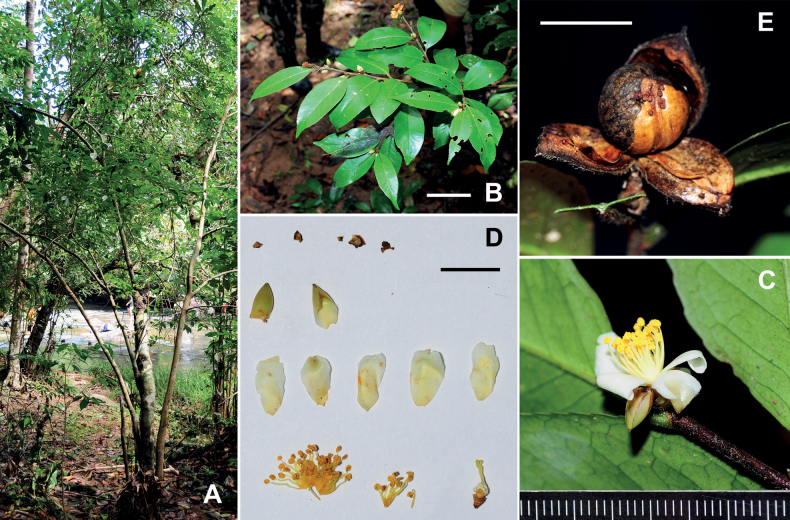
*Camelliakissi***A** habitat **B, C** branches with flowers **D** a dissected flower **E** a mature fruit with a single seed. Scale bars: 5 cm (**B**); 1 cm (**D, E**). The minimum graduation of the ruler in **C** represents 1 mm.

Shrubs or trees up to 9 m tall. ***New branchlets*** puberulous; ***terminal buds*** puberulous or glabrous. ***Petioles*** 2–7 mm long, puberulous; ***leaf blades*** elliptic, oblong or obovate, 3–13.5 × 1.5–5 cm, coriaceous, abaxially yellowish green and sparsely puberulous or glabrous, adaxially dark green, hirsute along midrib, midrib abaxially elevated and adaxially slightly impressed or flat, secondary veins 6–11 on each side of midrib, base attenuate, cuneate or rounded, margin serrate, apex attenuate, acuminate or caudate. ***Flowers*** 2–4.5 cm in diam., subsessile. ***Bracteoles*** and ***sepals*** 5–12, caducous, crescent to broadly ovate, 1.5–7 × 2.5–9 mm, abaxially glabrous, puberulous or pubescent, adaxially glabrous, margin ciliolate. ***Petals*** 5–6, white, elliptic or obovate, 8–25 × 4–17 mm, abaxially glabrous, puberulous or pubescent at apex, adaxially glabrous. ***Stamens*** numerous, 4–9 mm long; filaments yellow, glabrous, basally connate for 1–2 mm. ***Ovary*** globose, pubescent. ***Styles*** 3, basally connate, 2–7 mm long, basally pubescent and gradually becoming glabrous apically. ***Capsule*** globose or ovoid, 1–3 cm in diam., 1.5–3.5 cm in height, 1–3-loculed with 1–3 seeds; pericarp 0.5–1.5 mm thick. ***Seeds*** brown or black, hemispherical, polyhedral or globose, 1–2 cm in diam., glabrous Fig. 5.

##### Phenology.

Flowering October–February, fruiting March–August.

##### Distribution and habitat.

*Camelliakissi* occurs in evergreen and mixed deciduous forests, usually by streams, at elevations of 50–2150 m in Bhutan, Cambodia, China, India, Laos, Myanmar, Nepal, Thailand (Fig. 2), and Vietnam.

##### Additional specimens examined.

**Chiang Mai**: Chom Thong, Doi Inthanon, 3 December 1964, *Bunchuai 1420* (BKF SN060820), from 23 km point on the main road to Bang Nong Lum, ca. 1100 m, 16 February 1998, *Konta et al. 4312* (BKF); Mae Chaem, Doi Inthanon, 1700 m, 24 December 1996, *Hara A178* (BKF SN173304).

**Chanthaburi**: Soi Dao, Khao Soi Dao, ca. 1400 m, 13 December 1924, *Kerr 9651* (BK 203727, BM, C).

**Kamphaeng Phet**: Pang Sila Thong, Mae Wong, 900–1140 m, 14 June 1995, *Niyomdham et al. 4379* (BKF).

**Kanchanaburi**: Sai Yok, Chongkhaosoong, ca. 900 m, 12 August 1995, *Wongprasert s.n.* (BKF SN112557); Tong-pha-phum, E-tong, 28 January 2001, *Veesommai 1.38* (BKF SN145916).

**Khon Kean**: Phu Wiang, Tap Phaya Suea, 1 December 2003, *Mattapha 472* (KKU), 16°37'58"N, 102°12'59"E, 430 m, 22 December 2015, *Zhao et al. 137* (BKF, KKU, KUN, TCD).

**Loei**: Dan Sai, Phu Lom Lo, 1500–1600 m, 8 April 1922, *Kerr 5782* (BK 203732, BM, C); Phu Kradueng, ca. 1200 m, 12 March 1924, *Kerr 8695* (BK 203922, BM), 16°53'–54'N, 101°47'–49'E, 31 October 1984, *Mitsuta et al. T-40368* (BKF SN107567) & *T-42270* (BKF SN060860), Pha Nok En, 1 September 1988, *Fukuoka T-63791* (BKF SN103457), summit plateau, trail from Than Sawan to Tham Sor Nue waterfall, 1100–1200 m, 12 September 1990, *Chantaranothai et al. 90/143* (K); Phu Ruea, 1300 m, 4 May 1997, *Pooma 1550* (BKF), Phu Luang, 16 June 2004, Bunwong 296 (KKU), Khoknokkraba, ca. 950 m, 17 November 2000, *Wongprasert 11-13* (BKF SN133293); Wang Saphung, Phu Luang, 15 April 1968, *Chermsirivathana 899* (BK 203742).

**Nakhon Nayok**: Mueang Nakhon Nayok, Khao Yai, 14°26'10"N, 101°22'28"E, 730 m, 8 May 2003, *Charoenchai & Phomphuang 399* (BK 263128, CMUB 26064); Pak Phli, Khao Yai, 14°21'N, 101°22'E, 1200–1300 m, 29 October 1970, *Charoenphol et al. 4205* (BKF SN060889).

**Nakhon Ratchasima**: Pak Chong, Khao Yai, ca. 600 m, 11 August 1974, *Maxwell 74-786* (BK 203724), Orchid waterfall, 600–750 m, 18 November 1982, *Koyama et al. T-30008* (BKF SN060882), Haew Suwat waterfall, 700–730 m, 19 November 1982, *Koyama et al. T-30110* (BKF SN060881), 14°26'10"N, 101°22'29"E, 730 m, 7 October 2002, *Charoenchai & Phomphuang 343* (BK 263207, CMUB 26356).

**Nakon Si Thammarat**: Lan Saka, Khao Luang, 950–1000 m, 25 May 1968, *Beusekom & Phengkhlai 1002* (BKF SN060906, C).

**Nan**: Pua, Doi Phukha, 1700 m, 26 June 2008, *Pooma & Tamura 7113* (BKF SN188552).

**Phang-Nga**: Khao Pawta Luang Keow, 900–1000 m, *Geesink et al. 7690* (BKF SN060902).

**Phetchaburi**: Kaeng Krachan, Panoen Thung Ranger Substation, 12°52'12"N, 99°22'12"E, 123 m, 26 January 2005, *Williams et al. 1112* (K), summit of Khao Phanoen Thung, 12°52'5"N, 99°22'20"E, 1240 m, 8 May 2005, *Middleton et al. 3271* (BKF SN168267, E 00226065).

**Phitsanulok**: Chat Trakan, Phu Soi Dao, 1600 m, 14 August 2000, *Suksanthan 2684* (QBG 19436); Nakhon Thai, on the way to the headquarters of Phu Hin Rong Kla National Park, 16°57'5"N, 101°1'24"E, 1600 m, 9 November 2015, *Zhao et al. 71* (BKF, TCD).

**Prachin Buri**: ca. 1000 m, 4 July 1924, *Kerr 10824* (BK 203739); Khao Yai, at the Heo Suwat Falls, 750 m, 8 July 1966, *Larsen et al. 99* (C).

**Ranong**: 50 m, 17 November 1973, *Santisuk 597* (C); Muang Len, 160 m, 11 January 1966, *Hansen & Smitinand 11909* (BKF SN060905, C); Suk Samran, Khlong Na kha, ca. 9°45'N, 98°40'E, 50 m, 22 June 1974, *Geesink et al. 7381* (K) & *7404* (C, K).

**Sakon Nakhon**: Phu Phan, 19 November 1962, *Suwanakoset 1916* (BKF, K), ca. 30 km SW of Sakonnakhon city, ca. 380 m, 13 November 1984, *Murata et al. T-48771* (BKF SN060859), Huay Yai waterfall, 22 June 2003, *Chantaranothai et al. s.n.* (KKU); Sawang Daen Din, Ban Thon, 1 December 1962, *Adisai 194* (BK 203726).

**Si Sa Ket**: Kantharalak, Phanom Dongrak, 200 m, 25 November 2005, *Suddee et al. 2632* (BKF SN181662, QBG 29057), Sao Thongchai Phulaor Falls, Phanom Dongrak WS, trail to waterfalls, 200 m, 22 December 2005, *Pooma et al. 6068* (BKF SN183620, K).

**Surin**: Mueang Surin, Arloor-Doonban Community Forest, 124 m, 3 February 2008, *Petrmitr 840* (CMUB 29794).

**Udon Thani**: Na Yung, Phu Luang, 1050–1300 m, 8 January 1966, *Hennipman 3556* (BKF SN060870, C, K).

**Uttaradit**: Nam Pad, Phu Soi Dao, 1613 m, 17 November 2009, *Norsaengsri & Intamusik 6145* (QBG 42661).

##### Notes.

Wallich’s (1820: 429) original epithet of the species, *kissi*, cannot be replaced by “*kissii*” as shown in “Flora of China” (Ming and Bartholomew 2007), because it derived from the vernacular name (see Article 60 Ex. 21 of the Shenzhen Code, Turland et al. 2018; Zhao 2022c). *Camelliakissi* is the most widely distributed species of the genus in Thailand (Fig. 2). Edible oil can be extracted from its seeds (Baral and Acharya 1997), which may supply unique value in the breeding of oil camellias.

#### ﻿4b. Camellia kissi var. confusa

Taxon classificationPlantaeEricalesTheaceae

(Craib) T.L. Ming, Fl. Yunnan. 8: 300. 1997.

D8393636-E685-5206-BEE0-03EFAB914C4C

≡ Theaconfusa Craib, Bull. Misc. Inform. Kew (1): 5 1914. Lectotype (first-step designated by Craib [1925: 131]; second-step designated by Zhao et al. [2017a: 174]): THAILAND. Chiang Mai: Doi Suthep, 1200–1500 m, 31 October 1909, *A.F.G. Kerr 889* (K 000704304!).

##### Description.

**Figure 6.**
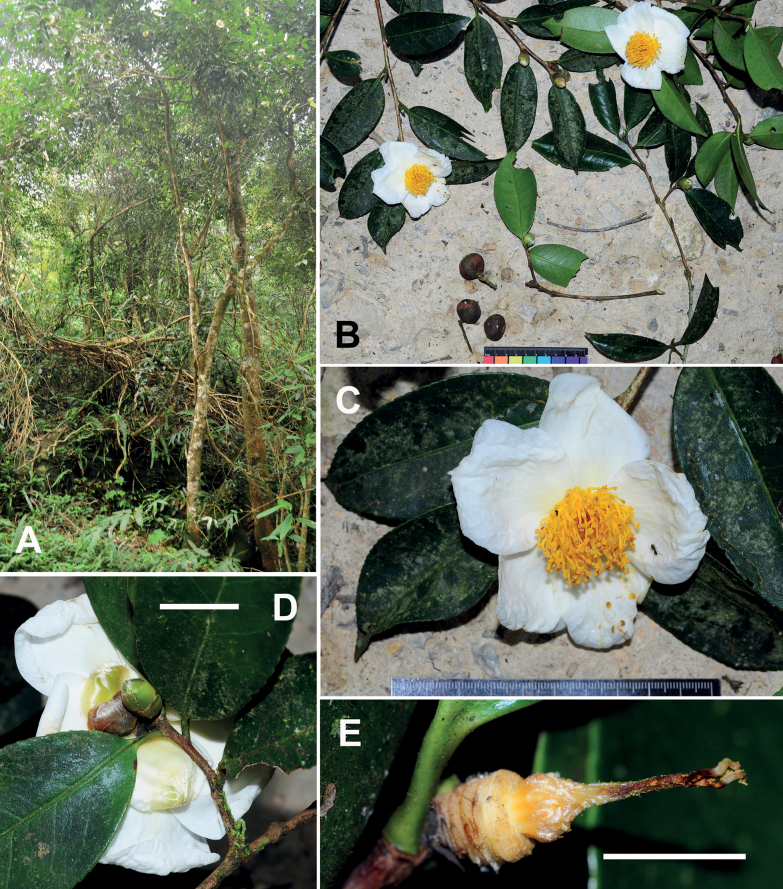
Camelliakissivar.confusa**A** habitat **B** branchlets with flowers, flower buds and caducous fruits **C, D** branchlet with flower **E** a pedicel and gynoecium. Scale bars: 2 cm (**D**); 1 cm (**E**). The minimum graduation of the rulers in **B, C** represents 1 mm.

Trees or shrubs up to 12 m tall. ***New branchlets*** puberulous; ***terminal buds*** puberulous or glabrous. ***Petioles*** 5–15 mm long, puberulous; ***leaf blades*** elliptic, obovate, oblong or ligulate, 5.5–13 × 2–4.5 cm, coriaceous, abaxially yellowish green and glabrous, adaxially dark green, hirsute along midrib, midrib abaxially elevated and adaxially slightly impressed or flat, secondary veins 7–11 on each side of midrib, flat or obscure on both surfaces, base attenuate, cuneate or rounded, margin sparsely serrate, apex attenuate, acute, acuminate or caudate. ***Flowers*** solitary, 4.5–10 cm in diam., subsessile. ***Bracteoles*** and ***sepals*** 6–8, caducous, crescent to broadly ovate, 1.5–20 × 3–10 mm, abaxially glabrous, puberulous or pubescent, adaxially glabrous, margin ciliolate. ***Petals*** 6–8 in 1–2 whorls, white, elliptic or obovate, 2.5–5.5 × 1.5–4 cm, abaxially glabrous, puberulous or pubescent at apex, adaxially glabrous, apex bilobed. ***Stamens*** numerous, 1–1.5 cm long; filaments yellow, glabrous, basally connate for 2–3 mm. ***Ovary*** globose, pubescent. ***Styles*** 3(–4), basally connate or distinct, 8–12 mm long, basally pubescent and gradually becoming glabrous apically. ***Capsule*** globose or oblate, 2.5–6 cm in diam., 2–5 cm in height, 1–3-loculed with 1–3 seeds; pericarp 2–8 mm thick. ***Seeds*** brown or fuscous, hemispherical, polyhedral or globose, 1.5–2.5 cm in diam., glabrous Fig. 6.

##### Phenology.

Flowering October–January, fruiting December–September.

##### Distribution and habitat.

Camelliakissivar.confusa is distributed in the evergreen forest at elevations of 350–1700 m in China, Laos, Myanmar, Thailand (Fig. 2) and Vietnam.

##### Additional specimens examined.

**Chiang Mai**: Chom Thong, Ban Pha Mon, 900 m, 31 May 1979, *Vidal & Vidal 6247C* (K); Doi Inthanon, 18°40'N, 98°25'E, 1400 m, 3 January 1975, *Geesink et al. 8045* (BKF SN060804, C), 1680 m, 19 December 1983, *Fukuoka & Ito T-35316* (BKF SN060813), 1700 m, 18 November 1998, *Hara et al. C002* (BKF SN173305) & *C044* (BKF SN173303), road to Khun Wang, 1310 m, 24 October 2000, *Chayamarit et al. 2204* (BKF SN173163); Fang, Doi Ang Khang, 1490 m, 8 December 1934, *Garrett 905* (BKF SN060834, E 00681092, K); Mae Cham, Mae Sanga, 1300 m, 6 May 2000, *Sangnin & Sillapasuwan 3346* (BKF SN138882), between 34 and 35 km, on the road to Mae Aum Watershed Management Station, 18°30'30"N, 98°30'19"E, 1636 m, 12 November 2015, *Zhao et al. 83* (BKF, KUN, TCD); Mueang Chiang Mai, Doi Suthep, ca. 1600 m, 22 December 1920, *Kerr 4680* (BK 203745), 1300 m, 20 February 1959, *Sørensen et al. 6967* (BKF SN060827, C, K), ca. 900 m, 17 November 1922, *Kerr 6676* (BK 203730, BM, K) & *6676A* (BK 203741, BM, E 00681089, K), 1050 m, 7 November 1987, *Maxwell 87-1379* (BKF SN060825); east side of Doi Pui at the Chang Kian Agricultural Station, 1350 m, 24 November 1988, *Maxwell 87-1351* (BKF SN060851); Samoeng, Samoeng Tai, Ban Pa Kar, 1424 m, 24 June 2008, *Jatupol 08-233* (QBG 35892).

**Loei**: Phu Kradueng, top area of Phu Kradueng, 1200–1300 m, 19 December 1982, *Koyama et al. T-31384* (BKF SN060872); Phu Ruea, 13 December 1966, *Umpai 311* (BK 203736); Phu Luang, 17°17'2"N, 101°31'8"E, 1440 m, 3 November 2015, *Zhao et al. 26, 27, 32* (BKF, KUN, TCD) & *33* (TCD); Wang Saphung, Phu Luang, 900–1000 m, 15 November 1968, *Chermsirivathana 1057* (BK 203722); 1410 m, 13 May 1998, *Chayamarit et al. 1357* (BKF).

**Nan**: Pua, Doi Phu Kha, 1100 m, 17 August 1995, *Pooma 1104* (BKF SN167083), 19°13'N, 101°5'E, 1500 m, 8 October 1998, *Srisanga 293* (QBG 12268) & *294* (QBG 12269).

**Sakon Nakhon**: Phu Phan, ca. 17°N, 104°E, 380–450 m, 12 November 1984, *Murata et al. T-51155* (BKF SN115022); Huai Lub Num Lud, 9 March 1996, *Puudjaa 195* (BKF SN091534).

**Sukhothai**: Khiri Mat, Kao Luang, ca. 1000 m, 2 May 1922, *Kerr 5917* (BK 203747).

##### Notes.

The variety usually bears a larger flower and a thicker pericarp than C.kissivar.kissi (Figs 5, 6; also see the key above). It is sometimes difficult to distinguish them because the size of flowers and fruits can vary gradually in the forests. However, the taxonomic treatment of two varieties suggested by Ming (2000) is provisionally followed here before more data, especially those of population genetics, are available.

#### ﻿5. Camellia laotica

Taxon classificationPlantaeEricalesTheaceae

(Gagnep.) T.L. Ming, Acta Bot. Yunnan. 21(2): 153. 1999.

08888844-CD2E-5830-B497-73801AD19301

≡ Thealaotica Gagnep., Notul. Syst. (Paris) 10: 128. 1942. Lectotype (first-step designated by Sealy [1958: 222]; second-step designated by Zhao et al. [2017a: 176]): LAOS. Savannakhet: entre Lang a xinhxa ne et L. xoan, 10 April 1927, *E. Poilane 13693* (P 04511456! Image: https://science.mnhn.fr/institution/mnhn/collection/p/item/p04511456).

##### Description.

**Figure 7.**
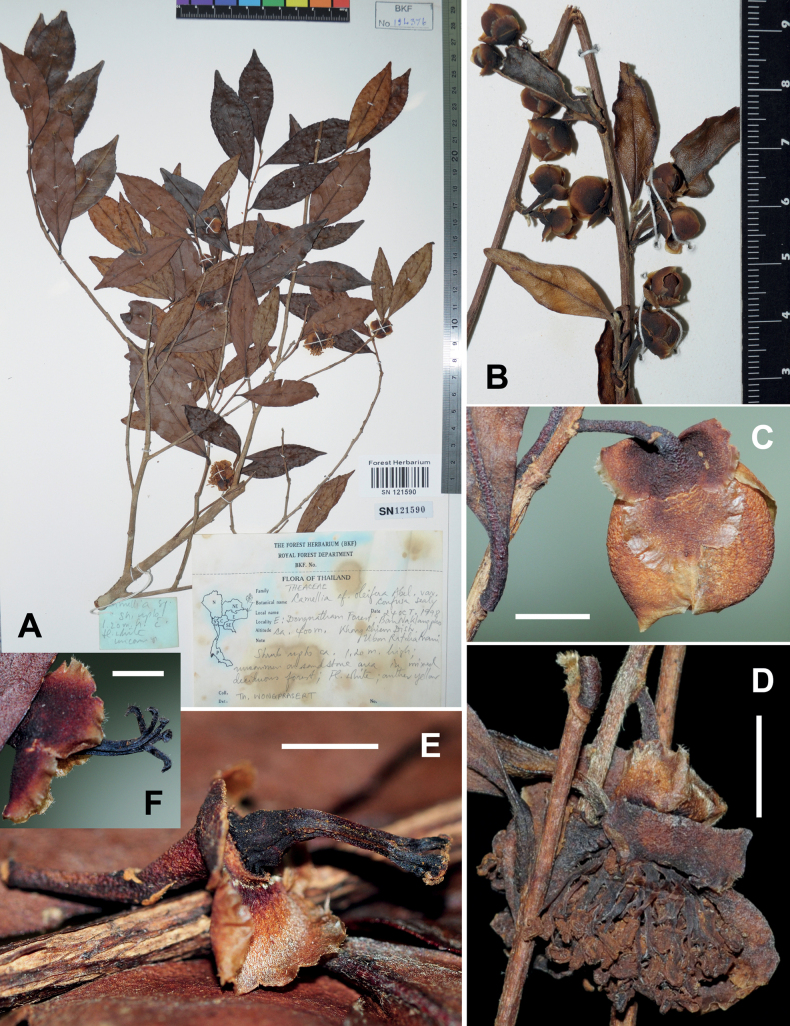
Dry specimens of *Camellialaotica*, *Wongprasert s.n.* at BKF **A** specimen sheet **B** branchlet with flower buds **C** a flower bud **D** a flower **E, F** flower fragments showing sepals and gynoecia. Scale bars: 5 mm (**C, D**); 3 mm (**E, F**). The minimum graduation of the rulers in **A, B** represents 1 mm.

Shrubs up to 5 m tall. ***New branchlets*** puberulous; ***terminal buds*** pubescent. ***Petioles*** 2–5 mm long, puberulous; ***leaf blades*** elliptic to oblong, 4–9 × 1–4.3 cm, thinly coriaceous, abaxially sparsely appressed pubescent, especially along midrib, adaxially glabrous, midrib and secondary veins abaxially elevated and adaxially channelled, secondary veins 6–8 on each side of midrib, base attenuate, margin sparsely serrulate, apex attenuate to shortly caudate. ***Flowers*** solitary or up to 3 in a cluster. ***Pedicels*** 7–14 mm long, glabrous or puberulous at base. ***Bracteoles*** 2, opposite or alternate, caducous. ***Sepals*** 3–4, persistent, sub-orbicular or broadly ovate, 3–8 × 6–9 mm, abaxially glabrous, adaxially sericeous, margin ciliolate. ***Petals*** 5, white, broadly ovate to obovate, 8–12 × 5–7 mm, glabrous on both surfaces, margin ciliolate. ***Stamens*** numerous, 5–7 mm long; filaments glabrous. ***Ovary*** ovoid, glabrous. ***Style*** 1, 5–7 mm long, glabrous, apically 3–5-lobed for 1–2 mm. ***Capsule*** globose, 12–20 mm in diam.; pericarp 2–3 mm thick. ***Seeds*** glabrous when immature Fig. 7.

##### Phenology.

Flowering October, fruiting April–August.

##### Distribution and habitat.

*Camellialaotica* is distributed in the evergreen or mixed deciduous forests at elevations of 400–750 m in Laos and Thailand (Fig. 2).

##### Additional specimens examined.

**Ubon Ratchathani**: Khong Chiam, Ban Na Klang Pho, Dong Na Tham forest, ca. 400 m, 24 October 1998, *Wongprasert s.n.* (BKF SN121590, BKF SN121591; Fig. 7).

##### Notes.

Gagnepain (1942) described the species based on three gatherings, including *Poilane 13268*, *13693* and *13743*. Sealy (1958) suggested that *Poilane 13268* and *13693* represented *C.laotica* whereas *Poilane 13743* was a distinct species, which was subsequently given a name, *C.sealyana* T.L. Ming by Ming (1999). Sealy (1958) and Ming (2000) supplied a short description of *C.laotica*, without the characters of flowers, based on two gatherings (*Poilane 13268 & 13693*) in Laos. I add an account of flowers and provide a detailed description above.

*Camellialaotica* is a new record to Thailand. It is represented by a single collection, *Wongprasert s.n.* at BKF (Fig. 7). The specimen was collected in Eastern Thailand, along Thai-Laos border (Fig. 2). The leaves of *C.laotica* in Thailand seem to be narrower than those of the syntypes (*Poilane 13268 & 13693*) from Laos, and they do not bear cork-warts on the abaxial surface by which *C.sealyana* (cork-warts present) can be distinguished (Fig. 7).

#### ﻿6. Camellia sinensis (L.) Kuntze var. assamica

Taxon classificationPlantaeEricalesTheaceae

(Royle ex Hook.) Steenis, Fl. Scholen. Indon. 280. 1949.

E38F4E45-B533-544F-9493-56E91C6101FC

≡ Theaassamica Royle ex Hook., Kew Gardens 28. 1847. Neotype (designated by Mabberley [2021: 1354]): India. Assam, January–February 1836, *W. Griffith s.n.* (K 000939670!).
= Thea cochinchinensis Lour., Fl. Cochinch. 1: 338. 1790. Neotype (designated by Zhao et al. [2017b: 1453]): Vietnam. Yen Bai: Bao Ha, 21 February 1936, *E. Poilane 25282* (P 04511587! Image: https://science.mnhn.fr/institution/mnhn/collection/p/item/p04511587).
= Camelliatheifera Griff., Trans. Agric. Soc. India. 5: t. C. 1838. Lectotype (designated by Zhao et al. [2017b: 1453]): India. Upper Assam, *W. Griffith s.n.* (TCD 0017977!).
= Thea yersinii A. Chev. ex Gagnep., Fl. Indo-Chine [P.H. Lecomte et al.], Suppl. 1: 310. 1943 (“Thea yersini”). Lectotype (designated by Zhao et al. [2017b: 1453]): Vietnam. Khanh Hoa: Massif du Hὸn bà, province de Nhatrang, 1000–1500 m, 12 September 1918, *A. Chevalier 38684* (P 02142599! Image: https://science.mnhn.fr/institution/mnhn/collection/p/item/p02142599).
= Camelliamultisepala Hung T. Chang & Y.J. Tan, Acta Sci. Nat. Univ. Sunyatseni 23(1): 11. 1984. Holotype: China. Yunnan: Mengla, Xiangming, cultivated, 1050 m, 3 December 1982, *Y.J. Tan & S.C. Ma A31002* (SYS 00095167!).
= Camelliapolyneura Hung T. Chang & Y.J. Tan, Acta Sci. Nat. Univ. Sunyatseni 23(1): 10. 1984. Holotype: China. Yunnan: Luchun, Qimaba, in tea garden, 1400 m, 18 November 1982, *Y.J. Tan & S.C. Ma A26001* (SYS 00090671!).
= Camelliasinensisvar.kucha Hung T. Chang & Ping S. Wang, Acta Sci. Nat. Univ. Sunyatseni 23(1): 10. 1984. Holotype: China. Yunnan: Jinping, Tongchang, 1371 m, 11 November 1982, *B.H. Chen & Y.J. Yang A22003* (SYS 00095188!).
= Camelliatenuistipa Orel, Curry & Luu, Pursuit Hidden Camellias Vietnam China: 263. 2015. Holotype: Vietnam. Gia Lai: Kon Ka Kinh National Park, 22 Jan 2011, *H.T. Luu & Q.D. Nguyen KKK 221* (NSW 901734, image!).

##### Description.

**Figure 8.**
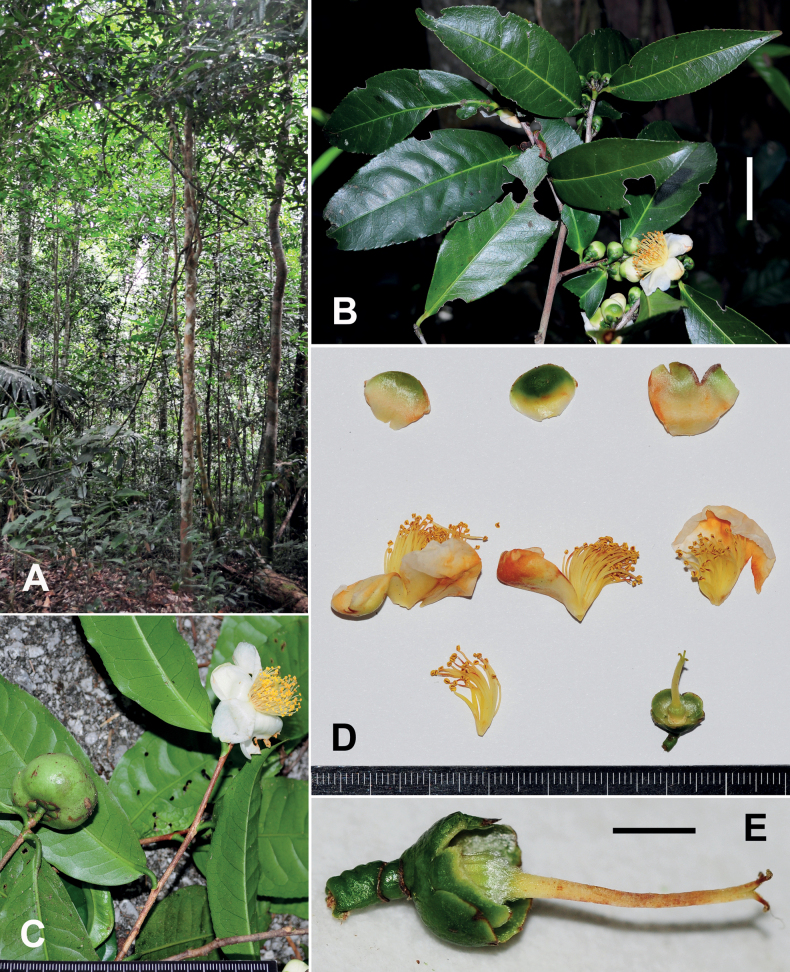
Camelliasinensisvar.assamica**A** habitat **B** branchlets with flowers and flower buds, adaxial surface of leaves **C** branchlets with flower and fruit, abaxial surface of leaves **D** a dissected flower **E** a flower without petals, androecium and a half sepal. Scale bars: 3 cm (**B**); 5 mm (**E**). The minimum graduation of the rulers in **C, D** represents 1 mm.

Trees or shrubs up to 20 m tall. ***New branchlets*** puberulous or pubescent, ***terminal buds*** pubescent or puberulous. ***Petioles*** 2–9 mm long, puberulous; ***leaf blades*** elliptic, oblong or obovate, 8–29 × 3.5–10 cm, coriaceous, abaxially yellowish green, puberulous, especially along midrib, adaxially dark green, shiny, midrib abaxially elevated and adaxially slightly raised or flat, secondary veins 7–15 on each side of midrib, abaxially slightly elevated and adaxially slightly impressed or flat, base cuneate or attenuate, margin serrulate, apex attenuate or acuminate. ***Flowers*** solitary or up to 4 in a cluster, 2–3.5 cm in diam. ***Pedicels*** 4–14 mm long, slender or gradually swollen towards the top, ***bracteoles*** 2–3, alternate, caducous. ***Sepals*** 5, persistent, sub-orbicular or broadly ovate, 3–4.5 × 3–5 mm, abaxially glabrous, adaxially sericeous, margin ciliolate. ***Petals*** 5–7 in 1–2 whorls, white or outer 1–3 petals with a tinge of green at apex, obovate, elliptic or rounded, 1–2.5 × 1–2 cm, glabrous on both surfaces. ***Stamens*** numerous in 3–5 whorls, 7–16 mm long, filaments pale yellow, glabrous, outer filaments basally adnate to petals for 1–3 mm. ***Ovary*** oblate or globose, pubescent. ***Style*** 1, 6–15 mm long, glabrous or gradually becoming glabrous upwards, apically (2–)3(–4)-lobed for 1–3 mm. ***Capsule*** oblate, bi-coccal or globose, 1.5–4.5 cm in diam., 1–2 cm in height, 1–3-loculed with 1–3 seeds; pericarp 0.5–1.5 mm thick. ***Seeds*** brown or fuscous, globose, hemispherical or polyhedral, 1.2–2 cm in diam., glabrous Fig. 8.

##### Phenology.

Flowering September–January, fruiting February–December.

##### Distribution and habitat.

Camelliasinensisvar.assamica occurs in the evergreen and semi-deciduous forests at the elevations of 200–2700 m in China, India, Laos, Myanmar, Thailand (Fig. 2) and Vietnam.

##### Additional specimens examined.

**Chiang Mai**: Chiang Dao, 25 November 1963, *Bunchuai 1359* (BKF SN060849, C, K); Khun Awn, 900 m, 30 January 1921, *Kerr 4726* (BK 203758, BM, K); Mae On, Huai Kaeo, trail along Mae Kampong Waterfall, 1100 m, 7 September 2011, *Pooma et al. 7791* (BKF SN196689); Mae On, Jae Son, 18°51'20"N, 99°22'1"E, 1500 m, 28 May 2011, *Pooma & Pattharahirantricin 7729* (BKF SN196803); Mae Taeng, Mae Taman, 1300 m, 27 September 1994, *Nanakorn et al. 1808* (QBG 1808); Mueang Chiang Mai, Doi Suthep, 1050 m, 7 November 1987, *Maxwell 87-1378* (BKF SN060836), 15 July 1988, *Maxwell 88-875* (BKF SN209981), 1 June 1993, *Maxwell 93-522* (BKF SN169943, BKF SN192202, CMUB 02747), 18 September 1995, *Kopachon s169b1* (BM, CMUB 07853).

**Chiang Rai**: Wiang Kaen, 600 m, 26 December 2009, *Pongamornkul 2806* (QBG 51200).

**Loei**: Dan Sai, Pu Lom Lo, 1500 m, 8 April 1922, *Kerr 5780* (BK 203759, BM, K); Phu Kradueng, 16°53'–54'N, 101°47'–49'E, 1150–1250 m, November 1984, *Murata et al. T-42519* (BKF SN060857) & *T-42796* (BKF SN060856), 1160–1180 m, 2 September 1988, *Fukuoka T-63807* (BKF SN103447); Phurea, Phuluang, 17°18'N, 101°30'E, 1070–1100 m, 4 November 2015, *Zhao et al. 37–41* (BKF, TCD); Na Haeo, Phu Suan Sai, 10 February 2004, *Pornpongrungrueng 441* (KKU), 1300 m,16 May 2006, *Maknoi 850* (QBG 27664), 14 May 2008, *Maknoi & Srisanga 2185* (BKF SN190334, QBG 38413), 2 September 2008, *Maknoi 2738* (BKF SN191010, QBG 40367), 3 September 2008, *Maknoi 2806* (BKF SN188351, QBG 40435), 17°30'N, 100°56'E, 1300–1330 m, 6 November 2015, *Zhao et al. 49, 50, 52 & 54* (BKF, TCD), *Zhao et al. 51 & 53* (TCD).

**Mae Hong Son**: Mae Sariang, Wat Chan, Ban Den, 988 m, 29 April 2014, *Norsaengsri 10930* (QBG 77549).

**Nakhon Sawan**: Khao Pado, 4 June 1922, *Kerr s.n.* (BK 203756).

**Nan**: Tha Wang Pha, 19°04'N, 100°40'E, 1100 m, 23 July 1992, *Larsen et al. 43513* (BKF); Song Khwae, Tham Sokoen, 19°23'3"N, 100°31'5"E, 1210 m, 30 November 2011, *Laongsri et al. 1957* (QBG 56767).

**Phayao**: Mueang Phayao, Doi Luang, 625 m, 19 November 1997, *Petrmitr 148* (CMUB 13047).

**Phitsanulok**: Chat Trakan, Phu Miang-Phu Thong, 800 m, 18 February 2010, *Romklao Botanical Garden 0005/2553* (QBG 59399); Nakhon Thai, Phu Hin Rong Kla, 16°59'38"N, 101°0'9"E, 1300 m, *Zhao et al. 72* (BKF, TCD); Phu Rom Rot, one of the peaks of Phu Miang, 1200–1600 m, 3 October 1967, *Shimizu et al. T-11514* (BKF SN060844, K) & *T-11515* (BKF SN060845), 1200–1650 m, 4 October 1967, *Shimizu et al. T-11653* (BKF SN060843, K).

##### Notes.

The nomenclature of C.sinensisvar.assamica has been clarified in recent studies (Zhao et al. 2017b; Mabberley 2021; a review in Chinese see Zhao 2022a). *Theaassamica* was not validly published in Masters (1844) but later validated in Hooker (1847). Steenis (1949), rather than Kitamura (1950), firstly proposed the name at new rank, C.sinensisvar.assamica. Zhao et al. (2017b) provided a detailed description of Assam tea, which is supplied above with adjustments to meet the requirements of a taxonomic revision here (Maxted 1992).

As an important resource of commercial tea, Assam tea is widely cultivated in the tropical areas of the world. Local Thai people collected natural seeds or seedlings and planted them around houses to use the new branchlets or leaves as a vegetable or fermented beverage (Khanongnuch et al. 2017; Zhao 2022a).

#### ﻿7. Camellia suddeeana

Taxon classificationPlantaeEricalesTheaceae

D.Wei Zhao, Phytotaxa 594: 232. 2023.

53DFAA15-42BD-583B-8905-4F287AE13201

##### Type material.

***Holotype***: Thailand. Nakhon Phanom: Ban Phaeng, Phu Lang Ka NP, trail to hill top, 17°58'58"N, 104°7'38"E, 373 m, 23 October 2015, *S. Suddee et al. 4981* (BKF SN235114!).

##### Description.

**Figure 9.**
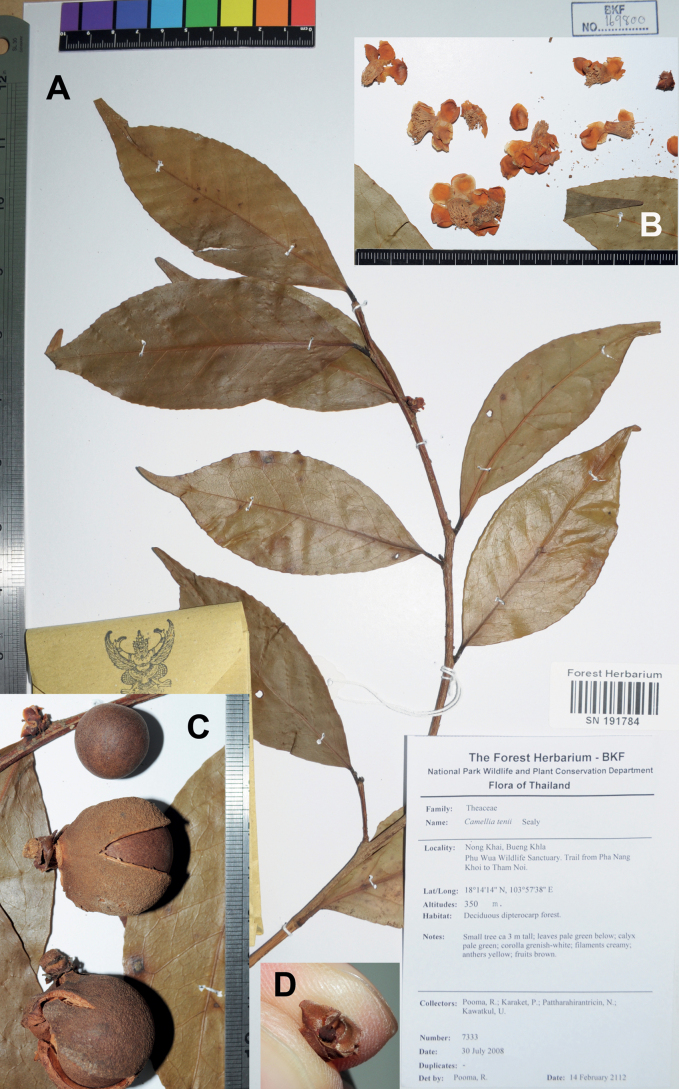
Dry specimens of *Camelliasuddeeana*, *Pooma et al. 7333* at BKF **A** specimen sheet **B** flower fragments **C** fruits and seed **D** calyx and gynoecium remains. The minimum graduation of the rulers in **А–C** represents 1 mm.

Shrubs or trees up to 10 m tall. ***New branchlets*** pubescent to villous, ***terminal buds*** puberulous. ***Petioles*** 2–10 mm long, pubescent or puberulous; ***leaf blades*** elliptic or oblong, 3.5–14 × 1.3–5.5 cm, coriaceous, abaxially dull green, sparsely appressed puberulous especially along midrib, or glabrous, adaxially dark green, glabrous or hirsute along midrib, midrib and secondary veins abaxially elevated and adaxially impressed, secondary veins 5–8 pairs, base attenuate, margin serrulate, apex acute, attenuate to caudate. ***Flowers*** axillary, solitary or paired, 1.5–3.5 cm in diam. ***Pedicels*** ca. 2 mm long. ***Bracteoles*** 3–6, persistent, deltate to semi-orbicular, 1–3 × 1.5–4 mm, abaxially glabrous or puberulous at apex, adaxially glabrous to slightly sericeous, margin ciliolate. ***Sepals*** 5–7, persistent, suborbicular, 2–5 × 4–5 mm, abaxially glabrous, adaxially sericeous. ***Petals*** 6–8 in 1–2 whorls, white, elliptic to obovate, 7–18 × 4–9 mm, abaxially glabrous, adaxially glabrous or sericeous at apex, apex obtuse to rounded, inner 4–5 petals basally adnate to filament whorl for ca. 2 mm. ***Stamens*** numerous, 6–10 mm long; filaments white, glabrous, outer filaments basally connate for 2–3 mm. ***Ovary*** globose to ovoid, glabrous. ***Styles*** 3(–4), distinct, 3–9 mm long, glabrous. ***Capsule*** globose or bi-coccal, 2.5–4.5 cm in diam., 1–2-loculed with 1–2 seeds; pericarp 0.5–2 mm thick, smooth or furfuraceous. ***Seeds*** light brown to fuscous, globose, 1.5–2.5 cm in diam., glabrous Fig. 9.

##### Phenology.

Flowering October–November, fruiting April–July.

##### Distribution and habitat.

*Camelliasuddeeana* is native to Thailand (Fig. 2) and occurs in the evergreen or deciduous forests at the elevations of 250–1200 m.

##### Additional specimens examined.

**Loei**: Ban Na Luang, 300 m, 20 November 1968, *Chermsirivathana 1185* (BK 203761, BKF SN060858). Phu Luang, ca. 1200 m, 23 November 1957, *Dee 1025* (BKF SN060875).

**Mae Hong Son**: Khun Youm, 8 April 1977, *Nimanong & Phusomsaeng 1816* (BKF).

**Nong Khai**: Bueng Khla, Phu Wua Wildlife Sanctuary, trail from Pha Nang Khoi to Tham Noi, 18°14'14"N, 103°57'38"E, 350 m, 30 July 2008, *Pooma et al. 7333* (BKF SN191784; Fig. 9); Phu Wua, 20 April 1996, *Niyomdham 4925* (BKF SN209980, BKF SN102858).

**Phetchabun**: Wang Thong, Thung Salaeng Luang National Park, 700 m, *Koyama et al. T-31930* (BKF SN060873).

**Pitsanulok**: Nahaew to Romklao Rd, 700 m, *Pooma 1237* (BKF SN090320).

**Uttaradit**: Nampad, Thud Phra Kiet National Park, ca. 650 m, 9 December 1994, *Santisuk et al. s.n.* (BKF SN109856).

##### Notes.

A specimen of *C.suddeeana*, *Chermsirivathana 1185* at BKF, were misidentified as *C.tenii* by Keng in 1970. *Camelliatenii* bears a smaller leaf (2.5–4.2 × 1.2–2.3 cm), villous ovary and pubescent seeds, whereas *C.suddeeana* has a larger leaf (3.5–14 × 1.3–5.5 cm), glabrous ovary and seeds (Fig. 9; Zhao 2023). The former is native to Yunnan, China and absent in Thailand but was included in Keng’s (1972) “Flora of Thailand”. As an essential element of a taxonomic revision (Maxted 1992), Zhao’s (2023) description of *C.suddeeana* is provided above with revisions.

#### ﻿8. Camellia taliensis

Taxon classificationPlantaeEricalesTheaceae

(W.W. Sm.) Melch. in Engler, Nat. Pflanzenfam. 2(21): 131. 1925.

F8A1945F-E87F-5814-88CB-80A0DB23E140

≡ Theataliensis W.W. Sm., Notes Roy. Bot. Gard. Edinburgh 10: 73. 1917. Lectotype (first-step designated by Ming [2000: 119]; second-step designated by Zhao et al. [2017a: 177]): China. Yunnan: Ghi Shan east of Tali Lake, 25°48'N, 2740 m, August 1914, *G. Forrest 13477* (E 00284542! Image: https://data.rbge.org.uk/herb/E00284542).
= Polysporayunnanensis Hu, Bull. Fan Mem. Inst. Biol. Bot. 8: 135. 1938. Lectotype (designated by Zhao et al. [2019: 299]): China. Yunnan: Lu-hsi Hsien, 1750 m, 6 February 1934, *H.T. Tsai 56805* (PE 00024542! Image: https://www.cvh.ac.cn/spms/detail.php?id=0756fff6).
= Camelliairrawadiensis Barua, Camellian 7(4): 18. 1956. Holotype: Myanmar. Raised from seed collected by L.O. Wilson, 1917, presumably in the region 26°–27°N, 98°–99°E (valley of Irrawadi in North Burma), January 1956, *Ex. Herb. I.T.A. 3253* (consisting of 2 sheets: K 000704313! & K 000704314!).
= Camelliapentastyla Hung T. Chang, Acta Sci. Nat. Univ. Sunyatseni 20(1): 92. 1981. Lectotype (designated by Zhao et al. [2018: 93]): China. Yunnan: Fengqing, cultivated, 2050 m, 12 February 1963, *L.F. Xia & Z.H. Yang 28* (KUN 1206061!).
= Camelliaquinquebracteata Hung T. Chang & C.X. Ye, Acta Sci. Nat. Univ. Sunyatseni 26(1): 20. 1987. Holotype: China. Yunnan: Lianghe, Dachang, 4 January 1983, *P. Zeng & Q.J. Xie 17055* (SYS, isotype: KUN 1206063!).

##### Description.

**Figure 10.**
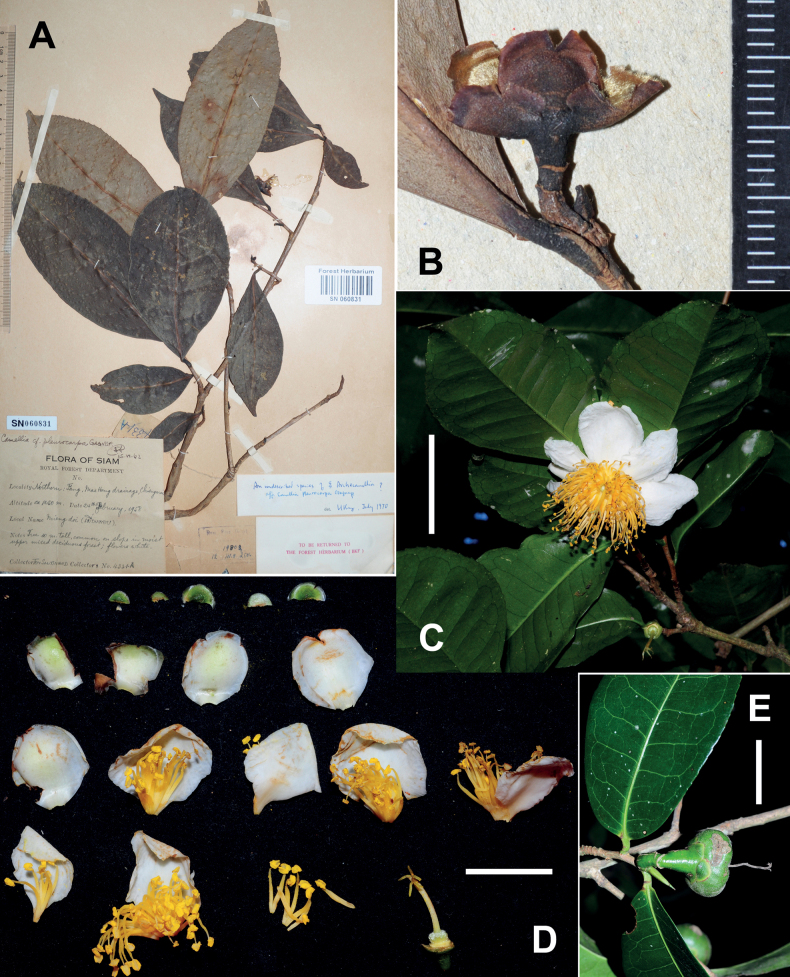
*Camelliataliensis***A, B** dry specimens of *Smitinand 4331A* at BKF **C** branchlet with flower **D** a dissected flower **E** branchlet with immature fruit. Scale bars: 3 cm (**C**); 2 cm (**D, E**). The minimum graduation of the rulers in **A, B** represents 1 mm.

Trees or shrubs up to 15 m tall. ***New branchlets*** glabrous, ***terminal buds*** glabrous or sparsely puberulous. ***Petioles*** 4–8 mm long, glabrous; ***leaf blades*** elliptic to oblong, 7.5–15.5 × 3–6.5 cm, coriaceous, abaxially yellowish green and adaxially dark or yellowish green, shiny and glabrous on both surfaces, midrib abaxially elevated and adaxially slightly raised, secondary veins 8–14 on each side of midrib, slightly elevated on both surfaces, base attenuate to obtuse, margin sparsely serrulate to nearly entire, apex attenuate or acute. ***Flowers*** solitary or up to 3 in a cluster, 3–5 cm in diam. ***Pedicels*** 8–15 mm long. ***Bracteoles*** 2–4, alternate, 2-ranked, caducous. ***Sepals*** 5, persistent, sub-orbicular or broadly ovate, 4–6.5 × 5.5–9 mm, abaxially glabrous, adaxially sericeous, margin ciliolate. ***Petals*** 7–11 in 2–3 whorls, white, elliptic to obovate, 16–30 × 10–21 mm, glabrous on both surfaces or inside sericeous at apex, inner petals basally adnate to filaments for 2–4 mm. ***Stamens*** numerous, 1–2.5 cm long; filaments pale yellow, glabrous. ***Ovary*** oblate, pubescent. Style 1, 11–20 mm long, basally sparsely pubescent and gradually becoming glabrous apically, apically (3–)5-lobed for 2–5 mm. ***Capsule*** oblate, 2.5–5 cm in diam., 2–3 cm in height,1–5-loculed with 1–5 seeds; pericarp 1.5–4 mm thick. ***Seeds*** brown, hemispherical or polyhedral, 1.5–2 cm in diam., glabrous Fig. 10.

##### Phenology.

Flowering October–February, fruiting April–November.

##### Distribution and habitat.

*Camelliataliensis* occurs in the montane evergreen forest at the elevations of 1100–2750 m in China, Myanmar and Thailand (Fig. 2).

##### Additional specimens examined.

**Chiang Mai**: Chiang Dao, Doi Chiang Dao, Den Ya Khat, 1500 m, 4 August 2007, *Watthana 2420* (QBG 30728); Chom Thong, Doi Inthanon, 1900 m, 1 May 1921, *Kerr 5298* (BK 203709, BM, K), 18°40'N, 98°25'E, 1700 m, 11 December 1969, *Beusekom & Phengklai 2462* (BKF SN061415, P 06838120), 1900–2025 m, 14 January 1994, *Fukuoka T-62168* (BKF SN102755); Fang, 1250 m, 24 February 1958, *Smitinand 4331* (BKF SN060803) & *4331A* ((BKF SN060831; Fig. 10A, B); Doi Ang Khang, 1700 m, 27 May 1998, *Wongprasert s.n.* (BKF SN121562); Doi Pha Hom Pok, 1400 m, 12 February 1958, *Sørensen 1607* (BKF SN060805, C, K), 1920 m, 1 February 2007, *Srisanga et al. 2919* (CMUB 29155, HITBC 143571, QBG 31244); Mae Ai, Bumuhn (Lahu) Village area, 1150 m, 21 October 1994, *Maxwell 94-1102* (BKF SN060837, CMUB 05366); Mae on, Ban Mae Kam Pong, 1300 m, 26 October 2007, *Pongamornkul 2121* (QBG 34196); Mae Taeng, 1600–1950 m, 5 December 1977, *Santisuk 1480* (C, K); Doi Chang, 1700–1900 m, 23 October 1979, *Shimizu et al. T-20524* (BKF SN060811, QBG 50044); Doi Kiew Lom, Huai Nam Dang, 1700 m, 18 January 2002, *Chayamarit et al. 3107* (BKF SN162068); Nanthaburi, Mae Tuen, 12 March 1991, *Smitinand s.n.* (BKF SN119850).

**Kampaeng Phet**: Klong Lam, Mae Wong, 1320 m, 30 October 2002, *Bult 609* (CMUB 20961).

**Mae Hong Son**: Khun Yuam, 20 November 1998, *Chusie KY308* (QBG 47303); Mae La Noi, Ban Dong, 1300 m, 15 December 2002, *Pongamornkul 1871* (QBG 35067), 2 May 2011, *Pongamornkul 2899* (QBG 64350); Mueang Mae Hong Son, along the trail to the peak of Doi Pui, 1100–1750 m, 16 December 2007, *Tanaka et al. HN8479* (QBG 35641); Doi Khun Huai Pong, 18°58'N, 98°10'E, 1850 m, 3 March 1968, *Hansen & Smitinand 12770* (BKF SN060846, C, E 00681068, K, P 04511721); Pai, en route to Doi Chang, 1800 m, 31 May 1972, *Santisuk 149* (BKF SN060809); Doi Mae Ya, 1800 m, 3 November 1999, *Suksathan 2030* (QBG 16229).

**Nan**: Pua, Doi Phu Kha, 19°17'N, 101°7'E, 1680 m, 10 April 1999, *Srisanga & Watthana 685* (QBG 14019), 19°10'N, 101°7'E, 1700 m, 26 May 2000, *Srisanga 1429* (QBG 17650), 1600 m, 22 August 2001, *Srisanga & Maknoi 2027* (QBG 21153).

**Phayao**: Muang, Doi Luang, 19°5'N, 99°27'E, 1500 m, 22 April 1998, *Sidisunthorn & Gardner 2568.0* (CMUB 13844); Phu Sang, Doi Pha Mon Noi, 19°44'8"N, 100°24'21"E, 1482 m, 30 April 2013, *Laongsri et al. 2849* (QBG 66589); Pong, Phu Lanka, 1500 m, 4 September 2006, *Watthana & Pumicong 2141* (QBG 28151).

**Tak**: Umpang, Umpang, near top of Khao Kheeo, 2150 m, 25 April 2001, *Bult 419* (CMUB 18159).

##### Notes.

Besides Assam tea, *C.taliensis* is another tea source plant that occurs in Thailand. Ming (1992) recognized *C.irrawadiensis* as a heterotypic synonym of *C.taliensis*. Chang et al. (1996) disagreed with Ming (1992) and suggested that *C.irrawadiensis* could be distinguished from *C.taliensis* by its abaxially punctate (vs. glabrous) and caffeine-free (vs. present) leaves, shorter pedicel (7–8 mm vs. 12–14 mm), larger sepals (5–7 mm vs. 2–4 mm) and smaller flowers (4 cm vs. 5–6 cm in diam.). Ming (2000) trivialized the differences and retained *C.irrawadiensis* in the synonymy of *C.taliensis*. The morphological differences between them listed in Chang et al. (1996) are, however, either inaccurate (e.g., the leaves of *C.irrawadiensis* are not abaxially punctate) or so trivial and overlapped (e.g., the size of pedicel, sepals and flowers) so that should be treated as an infraspecific variation. The claim of caffeine-free for *C.irrawadiensis* in Chang et al. (1996) was referred to Sealy’s (1958: 127) report. Sealy (1958) wrote that “…Dr. E.A.H. Roberts and Dr. D.J. Wood (both of the Indian Tea Association) tell me that chemically it [*C.irrawadiensis*] is clearly distinct from both, notably in that it does not contain caffeine.” Nevertheless, Nagata and Sakai (1985) analysed two samples of *C.irrawadiensis* and found that they contained 0.02% and less than 0.01% caffeine, whereas the content of caffeine in *C.taliensis* was 2.28%. However, the variation of chemical contents may be common in the plants of C.sect.Thea (L.) Griff. For instance, Ye et al. (1997) investigated 22 samples of *C.ptilophylla* Hung T. Chang and suggested that all individuals contained 0.57%–6.84% theobromine; and 18 of the samples were caffeine-free, while the remaining contained 3.02%–4.94% caffeine. Therefore, the single difference of the content of caffeine may be insufficient to warrant separation of *C.irrawadiensis* from *C.taliensis* at specific rank, and Ming’s (2000) treatment of them is followed here.

Keng (1972: 146) listed a single collection, *Smitinand 4331A* at BKF (Fig. 10A, B), under *C.pleurocarpa* in “Flora of Thailand”. However, the collection actually represents *C.taliensis*. *Camelliapleurocarpa* is native to Vietnam and bears an abaxially punctate leaf, 5–8 persistent bracteoles, abaxially sericeous perianth and glabrous ovary, whereas *C.taliensis* has a glabrous leaf, 2–4 caducous bracteoles, abaxially glabrous perianth and pubescent ovary.

## ﻿Discussion

Among nine taxa–seven species and two varieties–of *Camellia* in Thailand, only two species are endemic to the country: *C.connata* and *C.suddeeana*. As stated above, *C.pleurocarpa* and *C.tenii* in Keng’s (1972) account of *Camellia* are excluded from the flora of Thailand. The former was a misidentification of *C.taliensis* and the latter was frequently used to indicate *C.suddeeana*. *Camelliathailandica* was described based on a specimen collected from Thailand (Chang and Ren 1991); it is, however, a heterotypic synonym of *C.kissi* (Zhao 2022c). *Camelliaoleifera* C. Abel listed in Smitinand (1975) is actually *C.kissi* and the former is absent in the natural flora of Thailand. *Camelliasinensis* provided in Gardner et al. (2000) is merely cultivated in Thailand.

Most taxa of *Camellia* occur in northern and north-eastern Thailand (Fig. 2). This region geographically nears the current diversity centre of the genus—southern and south-western China and northern Vietnam (Chang 1981; Ming 2000). However, previous specimens were intensively collected from several provinces such as Chiang Mai, Loei, Mae Hong Son, Phayao, and Phitsanulok. Some under-collected gaps in the mountains of Chiang Rai, Mukdahan, Nan, and Nong Bua Lamphu could be searched in future to comprehensively understand the diversity of *Camellia* in Thailand (Fig. 2).

## Supplementary Material

XML Treatment for Camellia caudata

XML Treatment for Camellia connata

XML Treatment for Camellia furfuracea

XML Treatment for Camellia kissi

XML Treatment for Camellia kissi var. kissi

XML Treatment for Camellia kissi var. confusa

XML Treatment for Camellia laotica

XML Treatment for Camellia sinensis (L.) Kuntze var. assamica

XML Treatment for Camellia suddeeana

XML Treatment for Camellia taliensis

## ﻿Additional information

### Author ORCIDs

Dongwei Zhao https://orcid.org/0000-0002-7761-7127

### Data availability

All of the data that support the findings of this study are available in the main text.
